# Exploring the effects of a family admissions program for adolescents with anorexia nervosa

**DOI:** 10.1186/s40337-017-0181-z

**Published:** 2017-11-14

**Authors:** Keren Fink, Paul Rhodes, Jane Miskovic-Wheatley, Andrew Wallis, Stephen Touyz, Julian Baudinet, Sloane Madden

**Affiliations:** 1 Clinical Psychology Unit, the University of Sydney, Sydney, NSW 2006 Australia; 2 Department of Psychological Medicine, Department of Adolescent Medicine, Eating Disorder Service, The Children’s Hospital at Westmead, Sydney, NSW 2145 Australia

**Keywords:** Anorexia nervosa, Maudsley family based treatment, Adolescent, Qualitative research, Eating disorders

## Abstract

**Background:**

This study investigated patient experience in a Family Admissions Program (FAP) – a pilot treatment program for adolescents with Anorexia Nervosa at the Children’s Hospital, Westmead. Based on Maudsley Family Based Treatment (FBT), the FAP involves an adolescent and his/her family undergoing a two-week family-based hospital admission at the outset of treatment. The program aims to increase intensity and support to a level needed by some families struggling to engage with or access FBT.

**Method:**

Narrative Inquiry and Interpretative Phenomenological Analysis were used as a dual methodological approach to explore the prospective expectations and retrospective experiences of participants partaking in the program.

**Results:**

Results indicated that in cases where the family unit has been particularly fractured as a result of the eating disorder, the FAP offers an opportunity for relational strengthening and reunification. Combined with the program’s intensive support and proximity to hospital services, this serves to provide struggling families with enhanced skills and a stronger foundation for outpatient FBT.

**Conclusions:**

For families deemed at risk of unsuccessful outcomes with FBT, the FAP can be considered as an appropriate treatment adjunct to place alongside or before the commencement of FBT.

## Plain English summary

This paper looked at a novel adaptation of family based treatment (FBT) for adolescents with Anorexia Nervosa – the Family Admissions Program (FAP). The FAP involves a two-week stay in a residential hospital for the whole family of a patient who is struggling to get well with typical FBT.

Our research showed that the FAP was helpful for families who required more intensity and professional support in treatment. Being close to hospital services and supports during the FAP allowed members of these families to better develop skills to fight the illness.

A considerable number of individuals are expected to experience unsuccessful outcomes with typical FBT. This research offers important insights into how to develop more suitable adaptations of FBT for these patients and their families. This paper should be important to readers interested the areas of family therapy and eating disorders, particularly those hoping to learn about new avenues of clinical treatment in this field.

## Background

Research from the past three decades has shown that family-based treatment (FBT) for childhood and adolescent Anorexia Nervosa (AN) can often lead to positive outcomes at the end of treatment [1]. Clinical trials, systematic reviews and follow-up studies of FBT for adolescent anorexia nervosa (AN) [2] have demonstrated rates of remission between 31% and 49% 12 months after treatment, with improving rates of remission continuing to be seen for over five years following treatment [3–7]. Despite these findings, FBT does not yield favourable outcomes in a significant minority of cases. Studies examining moderating factors of treatment in clinical populations undergoing FBT found that complex family structures and characteristics, such as high parental criticism and high expressed emotion led to lower remission rates, early dropout and continuing symptoms post-treatment at follow-up assessment points [8–11]. Having a non-intact family unit was also shown to require longer and more supportive courses of therapy in these studies. Studies investigating the optimal length and frequency of FBT sessions [2, 12, 13] have also highlighted an association between more severe psychiatric psychopathology (such as higher rates of eating disorder symptomatology, as well as comorbidity) and worse treatment outcomes. In these cases, longer and more intensive administrations of FBT were required, with the researchers suggesting that more intractable presentations of AN are more challenging for parents to contain because behavioural and psychological change takes longer to be achieved.

To account for the treatment challenges posed by the above factors, a number of different adaptations to FBT have been developed over the past years [11, 14–16]. A central idea in many proposed adaptations for FBT has been to more directly address the aforementioned factors that may be maintaining AN symptoms within the family context, as well as focus on the possible need for higher levels of intensity and support in treatment that are often needed to drive important early therapeutic change for patients and families [17, 18]. Having early therapeutic change is especially important for families who exhibit the complex structures and characteristics previously described as leading to worse treatment outcomes, as well as for patients whose more complex illness psychopathology is likely to complicate the straightforward administration of FBT. Additionally, families who come from rural or remote areas with limited or no access to ongoing and regular specialised eating disorder services are likely to need more intensive doses of FBT in order for their treatment delivery to be commensurate with how manualised FBT is meant to be administered [2, 17].

In light of the existing limitations in treatment outcomes for particular individuals with AN, a FBT-based family residential program (Family Admission Program – FAP) has been piloted at the Eating Disorder Service (EDS) at the Children’s Hospital at Westmead, Australia (CHW), with the aim of targeting individuals and families who have struggled with FBT due to the aforementioned risk factors associated with poorer treatment. The FAP’s objective is to create a more intensive form of FBT, which involves the adolescent patient and his/her family living in a self-contained apartment within a hospital ward for a two-week period of daily treatment, often following a short inpatient admission for refeeding and medical stabilisation [17]. During this time, the family participate in a modified and condensed form of the first phase of FBT, but with additional interventions tailored to the family’s needs, such as managing distress or supporting meal time re-feeding skills. This occurs with multiple individual, sibling and family therapy sessions each week, and also multiple family meals. A comprehensive multi-disciplinary team is used to provide a higher level of medical and/or psychological support, providing the safety and containment necessary for parents to learn the skills to re-feed their child (see [17] for a detailed description of the structure, referral process and implementation of the FAP).

Despite the increasing use of the FAP at the CHW, no research exists evaluating its efficacy for the populations to which it targets. In light of this, the present study was largely exploratory in nature, aiming to investigate patients’ and families’ experiences during the FAP, and ask the question of what role participants felt the FAP had in their overall treatment for AN. Given the small number of participants available, a qualitative methodological approach was undertaken. This allowed for an open-ended and in-depth exploration of the phenomena in question, and also enabled richer, contextualised and more meaningful data regarding subjective experience to be elicited [19]. A secondary aim was to gain an understanding of how to improve the ongoing implementation of the FAP, and to guide future research evaluating the program’s intended treatment effects.

## Methods

The study comprised of two components: a Narrative Inquiry conducted with 10 families after they had completed the FAP; and an Interpretative Phenomenological Analysis (IPA) with 4 families and their clinicians, conducted both before and after they completed the FAP. This dual-methodological approach allowed for triangulation of the data, wherein phenomena in question could be studied via more than one methodology, and thus strengthen the validity of observed results [20]. These two components also complemented each other, since their combination offered the ability to have a dual set of data collection that could give a more comprehensive picture of participants’ experiences of the FAP at different time points in their overall treatment for AN [20, 21]. As a methodological tool, Narrative Inquiry provides a temporal framework for phenomena in question [22]. IPA, on the other hand, is concerned with personal interpretation of events and thus helpful in gaining an understanding of the subjective experience of the participant being studied [23]. For the current study, the Narrative Inquiry was thus expected to help place the effects of the FAP within participants’ overall treatment trajectories, whilst the IPA was used to capture a more detailed picture of specific and nuanced mechanisms of therapeutic change in the program. It was thus hoped that the IPA would complement the findings of the narrative inquiry by zooming in on how the features of the FAP actually worked to achieve change for participants.

A specific constructionist framework [24] was taken for the paradigms of inquiry, as it underscores the notion that meaning generated from research is inevitably a product of co-constructed knowledge between the researcher and participant. This paradigmatic approach thus allowed for the theoretical influences of the researchers to be acknowledged and incorporated into the collection and analysis of data. These influences predominantly included the core philosophical tenets of FBT, in which all the authors had clinical experience.

### Narrative inquiry.

A Narrative Inquiry [22, 24, 25] was first conducted to assess and explore the role that the FAP played in the history of AN in the family, as well as the meaning that the family ascribed to the program in the recovery process. Narrative inquiry involves the construction of narratives of personal experience by participants, wherein interviews between researcher and subject typically serve as the primary means of data collection. These are then transcribed and written as the narrative, which is then analysed for overarching themes of meaning. Narrative Inquiry provides a temporal context for investigation [22], allowing participants to assign meaning to past personal events [25]. It thus seemed an appropriate methodological tool for eliciting participants’ reflection on the meaning of the FAP within their overall journey through the illness.

#### Participants

In order to recruit as large a sample size as possible, non-purposive sampling was used, in which all families who had completed the FAP by the time that data collection commenced for the Narrative Inquiry were eligible for recruitment. Inclusion and exclusion criteria for participation in the study followed the same criteria used for participation in the FAP [17]: the participant was under 18 years of age with a primary diagnosis of an eating disorder; the participant had been medically stable (pulse 50 bpm, temperature > 35.5 °C, blood pressure > 80/40) for at least 72 h prior to admission to the FAP; the participant was eating a minimally sufficient intake for continuing medical stability.

A total of 14 families were contacted and sent information about the study. Three families declined to respond to the researchers after being sent the initial information, and so were not included in the study. One family declined to participate after being contacted by researchers. See Table 1 for details.

**Table 1:** Participant Demographics for Narrative Inquiry

Participant	Age	Family structure	Location	Complex presentation/Comorbidity	Participating family members in interview	Phone/Face-to-face interview
1 (female)	12	Intact	Rural	No	Parents, adolescent, all siblings	Phone
2 (female)	12	Intact	Inner metropolitan area	No	Parents and adolescent (no siblings in family)	Face-to-face
3 (male)	13	Intact	Outer metropolitan area	No	Parents, adolescent, all siblings	Phone
4 (female)	13	Intact	Inner metropolitan area	Yes: self-harm	Parents, adolescent, all siblings	Face-to-face
5 (female)	14	Non-intact	Inner metropolitan area	No	Parents, adolescent, all siblings	Face-to-face
6 (female)	14	Intact	Rural	No	Mother, adolescent, one out of two siblings	Phone
7 (female)	15	Intact	Interstate	Yes: self-harm; OCD	Parents, adolescent, all siblings	Phone
8 (female)	15	Non-intact	Inner metropolitan area	Yes: self-harm; complex trauma	Parents, adolescent (siblings not present)	Face-to-face
9 (female)	16	Intact	Outer metropolitan area	Yes: OCD	Mother, adolescent (siblings not present)	Face-to-face
10 (female)	16	Non-intact	Inner metropolitan area	Yes: self-harm; complex trauma	Parents, adolescent, all siblings	Face-to-face

#### Procedure

In-depth, semi-structured interviews were developed by the authors, in line with the research question. They were then conducted either by phone or face-to-face with each family with the first author. The interview questions asked the family to recount the story of their journey with AN to date, specifically focusing on their experiences during the FAP (interview schedules can be sought from authors upon request). Interviews lasted between 60 and 90 min and were audio-recorded and transcribed verbatim. The interview transcripts were then used to create an overarching narrative by the first author, which aimed to tell the story of how and where the FAP fit within each family’s experience responding to the challenges of AN. Narratives were created by first organising the data from each transcript temporally, beginning with the onset and course of AN, up to the present day, and then ending with family members’ reflections on the FAP [25]. Once the data was organised in this way, it was then written into a story that flowed coherently and contained a distinct beginning, middle and end. Upon completion of the narrative, it was reviewed by the family so that any revisions they deemed necessary could be incorporated into the story to ensure that their voices and experiences were authentically reflected [26]. Families were interviewed between 6 and 10 months following their completion of the FAP. At the time of their interview, four families were still undergoing the final phases of FBT and had largely achieved weight, eating and exercise behavioural containment. All participating families had thus reached similar stages in their trajectory of treatment.

#### Data analysis

Data analysis first involved an in-depth, line-by-line analysis of each completed narrative, where regularly occurring words and themes could be identified [22]. From this, provisional thematic categories were developed, which reflected the main themes and sub-themes running through each story. These were then compared and contrasted across cases, so that any themes or phenomena common to the participant sample could be identified. To maintain validity and rigour in the interpretation and analysis of data, cross-coding by the second author was performed with four out of the ten cases both at the beginning and end of analysis. This involved the second author going through the same analytic process, so that the final analysis yielded themes that had a reliable degree of agreement between coders.

### Interpretative phenomenological analysis (IPA)

The aim of the second component of the study was to achieve a more detailed analysis of families’ real-time experiences whilst they were currently undergoing the FAP, and then compare their real-time reflection of events to the clinical observations and thoughts of their treating clinicians. Since primary focus was on exploring factors within participants’ experiences, and the group comprised only a small number of cases, Interpretative Phenomenological Analysis (IPA) was used. IPA has been reported useful for such sample sizes [27] and is particularly committed to obtaining a detailed and rich interpretative understanding of personal experience of a phenomenon or event [23].

Each family’s treating pair of clinicians was also interviewed. The clinicians were either clinical psychologists or clinical social workers, who had been trained and practised extensively in FBT and family therapy.

#### Participants

Any families participating in the FAP at the time of data collection (during the year of 2014) were eligible for participation. A total of six families who underwent the FAP during 2014 were sent information about the study. The aforementioned inclusion/exclusion criteria for participation in the FAP were again used for participation in the IPA (see ‘Participants’ section of Narrative Inquiry). Two families who commenced but did not complete the FAP during this time were excluded from the study, so that a total of four families took part. For each of the four families, their two treating clinicians were also interviewed together as a pair. See Table 2 for details on participating families.

**Table 2:** Participant Demographics for IPA

Participant	Age	Family Structure	Location	Complex presentation/Comorbidity	Participating family members in interviews	Phone/Face-to-face
1 (male)	15	Non-intact	Interstate	No	Both parents, adolescent, all siblings	Face-to-face
2 (female)	15	Non-intact	Rural	Yes: self-harm; ASD	Both parents, adolescent, all siblings	Face-to-face
3 (female)	15	Intact	Inner metropolitan area	No	Both parents, adolescent, all siblings	Face-to-face
4 (female)	12	Intact	Outer metropolitan area	No	Both parents, adolescent, all siblings	Face-to-face

#### Procedure

Semi-structured interviews were conducted by the first author at the hospital, with the family alone and then with their treating pair of clinicians. Interviews were conducted at two time points: once on the first day of the FAP (pre-treatment) and once on the last day of the program (post-treatment). Pre-treatment interviews asked participants to discuss the reasons behind the family’s admission into the program, as well as any expectations of change. In the post-treatment interviews, participants were asked to reflect upon the family’s experiences of the program and any outcomes they witnessed in light of their initial expectations (questions in both sets of interviews were appropriately phrased to suit whether it was the family or clinicians being interviewed). Both sets of interviews involved a series of open-ended questions, which encouraged participants to talk in detail about their experiences. Each interview lasted for approximately 45–60 min. The interviews were then audio-recorded and transcribed verbatim, and the transcripts coded and analysed for themes that emerged both within and between cases.

#### Data analysis

Data analysis was performed by the first author and began with an iterative, in-depth analysis of each set of interview transcripts [27]. Detailed codes of meaning were generated from the data, which aimed to interpret and capture the meaning that participants made from their experiences in the FAP. Recurring codes were then catalogued and connected together, so that a pattern of themes of experience of the FAP could be developed and compared across cases. The final set of themes and super-ordinate themes were formulated into a table, where evidential instances of each theme could be located in the transcript [27]. Again, rigour and validity in the interpretation and analysis of data was attained by the second author cross-coding two out of the four cases.

## Results

### Narrative inquiry

Family members’ reflections on the role of the FAP highlighted a common set of experiences that could be conceptualised into five predominant themes, from onset to recovery: 1) a progressive intensification of AN; 2) fracturing of the family unit; 3) reaching a crisis point; 4) participation in the FAP; and 5) ongoing process of recovery. See Table 3.

**Table 3:** Narrative Synthesis: From onset to recovery

Theme	Experience
Progressive Intensification of AN	Quick escalation of symptoms
	Series of failed treatment attempts and/or hospital inpatient admissions
	Unsuccessful engagement with FBT
Fracturing of the family unit	Parents differing in opinion on severity, nature and appropriate treatment of illness
	Family members each feel isolated
	Family unit disconnected
Crisis point reached	AN reaches extreme level
	Family unit disconnected
	Family at ‘rock-bottom’
	FAP accepted as last-resort option
Participation in FAP	Re-integration of family unit
	Positive experience overcoming AN
	Insight and motivation to fight AN
Ongoing process of recovery	Re-engagement with FBT
	Ongoing process of improvement from AN, with periods of relapses and remissions

#### Progressive intensification of AN

For the majority of families, parents reported being taken aback by the seriousness and rapid escalation of their child’s eating disorder symptoms –[Mother]: *“I didn’t see the beginnings of the anorexia for a while…We just didn’t understand what it was”*. Nearly all of the families reported a long period of trying to resolve the disorder themselves, with little success. The inability to contain the growing intensity of the AN symptoms led parents to eventually seek out more specialised assistance; however, factors such as remote living locations and severe symptomatology prevented a lot of families from accessing adequate services when needed, leaving them to feel particularly hopeless and isolated from effective supports.

No family reported successful engagement with FBT, despite being linked in with CHW by this time. Families were either unable to access services which offered eating disorder treatment (often because of their remote living location), or they struggled to engage in the therapy effectively. This was a result of a number of issues including family members being unable to agree to FBT treatment structure as planned, or because an unexpected inpatient admission meant family sessions had to be suspended.

#### Fracturing of the family unit

All participants reported increasing disconnection within the family unit as family members struggled with the rising intensity of the AN. Within the parental subsystem, there was often a split between parents regarding the seriousness of their child’s AN, causing them to feel increasingly divided and alienated from each other, particularly in their approach on how to address and treat the disorder – [Mother]: *“my husband just did not really understand what was needed…so we would end up fighting…he’d say, ‘You’re making this a bigger issue. I don’t see any issue’, and I’m thinking, ‘Are you seeing anything!’”* Siblings similarly reported feeling distanced from the family unit, largely because of their desire to avoid the growing household strain and conflict.

*Crisis point reached.* Eventually, the effect of the family’s sense of disconnection and despair, combined with the extreme presentation of AN, resulted in them reaching a crisis point. By this point, families felt that they had exhausted all feasible treatment options. Some of them had been offered the opportunity to participate in the FAP at previous points, but turned it down due to how disruptive they expected it would be. In other cases, it had not yet been considered a necessary step to be offered by the treating team at CHW. Nonetheless, the sense of peril experienced at the time of crisis prompted all families to agree to participate in the FAP, which they viewed it as their last remaining option *–* [father]: *“but then eventually… when we hit that big low, we all just decided, ‘Okay, this is pretty much our last option, otherwise we’ve got no idea what else we can do”*.

#### Experiences during the FAP

All families described the two-week period of the FAP as highly intensive. They attributed this to the number of therapy sessions, family meals and activities each day, as well as the experience of everyone living together in the small self-contained apartment for the whole program. A ‘hothouse’ effect of therapeutic change was produced, in which families described being unable to avoid confronting issues that had been previously sidelined **–** [adolescent]: *“When you’re doing it every day, you can be directly asked questions about all the issues that you probably knew were there, but were too hard to discuss”.* Coming face to face with the full extent of behaviours associated with the AN also meant that family members saw the severity of the disorder in the same way for the first time. Not only did this unify an understanding of what AN was and how it had affected their family, but it also gave them a shared experience of dealing with the disorder that could begin to bridge the gaps that had developed in different family relationships –[Mother]: *“It made [father] understand a little bit more about the illness, about what I had been dealing with, and it helped me understand about him feeling left out, shut out. So I think our relationship particularly benefited”*. In addition to these relational changes, experiencing even a minor triumph or positive experience in overcoming the AN seemed to give parents motivation and confidence in continuing the principles learned during the FAP once back at home – [Father] “*and in the end…after succeeding on that food penalty plan* [a behavioural contingency management system]*…there was a breakthrough… and that changed stuff for us at home”*.

#### Ongoing process of recovery

The time following participation in the FAP was characterised by a gradual movement away from the AN. During this period, there was also a re-engagement with FBT from eight out of the ten families, either at CHW or at a service closer to their home. Families described recovery as an ongoing process that they were still working towards (one which included the management of both mental health and AN-related issues), rather than a discrete stage at which they had arrived – [Mother] “*There’s always stuff that’s happening with her and [the AN] is still continual. It’s still ongoing…you can’t not turn on for a day”*.

### Interpretative phenomenological analysis

The IPA highlighted six major themes of participant experience during the FAP, which summarised both the family and clinical aims of participating in the program, as well as their final evaluations of the program’s effects and outcomes. See Table 4.

**Table 4:** Themes of experience in IPA

Pre-treatment	Post-treatment
*Complexity requiring FAP*	*Changes towards the AN*
*Family’s hope for broad change*	*Changes in the family*
*Clinicians’ need for further assessment*	*Changes in the adolescent*

### Pre-treatment

#### Complexity requiring FAP

Prior to commencing the program, families and clinicians both spoke of complexity in the adolescent’s illness presentation and family context (such as a remote living location and/or non-intact family structure) as being primary reasons prompting the family’s consideration for the FAP, particularly because such factors were expected to limit the family’s ability to effectively engage in FBT long-term – [clinician]: *“She wasn’t eating or drinking anything in the week prior to admission, and when she was [an inpatient] she was actually trying to keep the NG tube in as much as possible, saying things like I don’t want to go home... which is different to typical presentations”*.

#### Family’s hope for broad change

Families’ reasons for participating in the program reflected their hopes for bettering their chances of change, not just in regard to the eating disorder symptoms, but also in terms of a wider change in the adolescent and family unit. They looked at it as a transitional step that could help them feel more skilled and prepared at challenging illness behaviours prior to returning home – [mother]: *“I think we were a little bit anxious about going straight into outpatient treatment… that we all weren’t ready for that. So we can now be in a setting where…we are given the reign, but also given some help if we need it”*.

#### Clinicians’ need for further assessment

For the clinicians, the need for further assessment of the family unit, prior to commencing weekly outpatient treatment, was seen as important for a positive treatment experience, and thus made them the view the FAP a necessary treatment step in better consolidating ongoing FBT for these families.

### Post-treatment

#### Changes toward the AN

For the majority of participants, their reflections following the FAP seemed to satisfy their initial expectations and desires for change. Importantly, the outcomes described by participants suggested an overall shift in the way that the family approached the AN and their situation, rather than a change in the illness itself. Whilst there was a reduction in eating disorder symptoms reported in all four families, this was not necessarily the primary point of improvement reflected upon by participants.

#### Changes in the family

Both clinicians and families observed changes in the way that family members viewed and related to the disorder, particularly in terms of parents feeling more confidence and effectively skilled in their capacity to manage eating disorder behaviours, as well as having a better understanding of the reality of what ongoing FBT would entail. Changes in family functioning and interactional dynamics were also noted by both clinicians and family members, predominately in regard to the way that family members related to and communicated with each other, as well as in the way that the family reorganized itself against the AN – [mother] *“we devoted a lot of the time to getting our, sort of system, working properly so we don’t drop things through the cracks”*.

#### Changes in the adolescent

Finally, for three out of the four families, some sort of wider shift within the adolescent and his/her demeanour and personality was seen. This was observed either in the way that the adolescent related to and engaged with the rest of the family, or in their behaviour and level of distress in regard to eating – [sibling]: *“she talked about wanting to get a babysitting job…and it means that she’s thinking about a more positive life… she has meaning.”*

### Key factors of the FAP connected with post-treatment outcomes.

The IPA importantly highlighted two factors that were consistently noted across all four cases as being especially necessary in leading to the observed outcomes at the end of the FAP: a) intensity, and b) proximity to supports.

#### Intensity

The high frequency of sessions and self-contained family living arrangement was seen to enhance treatment intensity in a positive way. Participants explained that the increased number of sessions over the short two-week period allowed for more opportunities than normal to discuss and work through family issues that would have otherwise been put aside in the process of re-feeding – [father]: *“if you’re coming in here for weekly sessions this would take months and months and months… because if you’re having problems one day and it was going to be another few days until your appointment, you might not have been able to recover quite as well or work through it”*. The clinicians elaborated on this point, adding that the increased face-to-face time with the family not only allowed for further assessment (which could then feed back into the direction of sessions), but it also sped up the structural change within the family unit that they were trying to achieve. The family’s intensive living arrangement during the program similarly created more opportunities for change, in which there was a sense of ‘induced bonding and interaction’ as a result of having to live together for two weeks without any of the typical distractions and responsibilities of daily life – [father]: *“All the other distractions of work and school and the things that need to get done at home, just haven’t been here”*.

#### Proximity to supports

The close proximity to hospital supports including after-hours support and the hospital emergency department also allowed parents to have a more comprehensive idea about the full array of strategies and resources which existed and could be used if needed, particularly in extreme cases. Parents noted that this proximity also made them feel confident enough to implement and persist with these strategies, that they would have otherwise felt were too risky or extreme at home, such as calling the police or an ambulance in response to food refusal or dangerous behaviour. In addition to having access to the whole team, this created a sense of containment and safety, which seemed to further enable processes of therapeutic change to take place more easily – [father]: *“we then really clearly understood how far we could push and what resources were around us to enable [her eating] to happen”*.

## Discussion

The objectives of this study were twofold: first, to explore participants’ experiences and processes of change during their time in the FAP; and second, to use these insights to establish possible areas of improvements of further implementations of the program, as well as to inform paths for future research of treatment effects. The two methods identified a number of common themes in the families’ experiences during the FAP. Illness complexity and a desire to remediate negative impacts on family functioning were identified as reasons that families first sought to engage in the FAP, whilst structural changes in the family unit and its enhanced response to managing the AN, were seen as key positive outcomes at completion of the program. Importantly, these factors of change were also endorsed by clinicians. The confluence of themes from the two methods of qualitative analysis thus added to the strength of findings in addressing the study’s objectives. Taken together, these findings highlighted four overall factors that led to change for participating patients and families.

First, the family unit appeared to reconnect and restructure itself in a way that was more effective at meeting treatment goals during the FAP. This was largely due to the adoption of more clearly delineated family roles, as well as relational styles that no longer avoided conflict. It thus seemed that the program allowed for the development of more positive family relationships, which had a protective quality that helped the family start to change longstanding unhelpful patterns of interaction. This also reflects previous findings that these structural and relational characteristics are important in effectively containing and reducing AN behaviours [2, 28, 29]. It thus seemed that the FAP helped families in this study reunify and emerge from a point of crisis, which ultimately resulted in the re-engagement with FBT for 8 out of 10 of the families in the Narrative Inquiry. This suggests that the program could help other families at risk of poor prognoses improve their responses to treatment. A suggested mechanism of change in FBT has been to increase anxiety to mobilise action; however, here change was initiated by increasing support equal to the crisis faced [2]. This may be an important adjustment to further explore, as some families may need treatment to be organised differently in order to benefit from the initial FBT goals.

Second, the presence of even a small change to managing the AN was shown to lead to a renewed sense of motivation for ongoing treatment for nearly all of the families. Such successes allowed families to challenge feelings of inadequacy and hopelessness about their child’s illness. Focusing on how to initiate these types of changes appears to be a key aspect in helping families become unstuck. It is likely that the FAP creates an important type of supportive environment – one that allows families to practically do something different, but also to recognise that an exception to their previous approach is occurring. This then reduces the negative impact of their previous failures, whilst also amplifying feelings of hopefulness [30, 31].

Third, the environmental context of the FAP was important in mobilising parents’ efforts at change at the same time as making them feel supported. This was largely through the immediate and proximal access to hospital-based services, which seemed to provide families with a sense of containment and reassurance. For example, the closeness to services such as the emergency department and after-hours psychological and psychiatric interventions helped parents feel confident about resorting to more extreme, but ultimately necessary, measures much earlier than would have normally been the case. This suggests potential benefits from having the family reside within the hospital, rather than at a separate location. The intensive design of the FAP was a similarly key environmental factor for participants, namely the presence of multiple daily therapy sessions, as well as the family residing together in the hospital for the duration of the program. Indeed, the benefits of intensive therapeutic engagement such as this have been historically noted in a number of other therapeutic disciplines, where the more intensive frequency of sessions has been shown to prevent opportunities for avoidance and other defences to emerge and derail treatment progress [32, 33].

Finally, the fact that families and clinicians had analogous expectations and evaluations of the FAP suggests that the two groups understood the treatment needs in a similar way. It seems that this mutual understanding could help to create a stronger therapeutic alliance between parents and clinicians which could then also add to parents’ sense of support and efficacy [34]. Indeed, previous research has highlighted that there are therapeutic benefits of commonly shared treatment goals and alignment between therapists and families [35–38].

## Conclusions

In light of the growing research showing that a considerable number of families will struggle with FBT [3, 18, 39] the results of this study offer potential insights into how to administer a program such as the FAP. The current results suggest that for families who are coming to treatment resource-poor, under relational strain and at an acute point of crisis, a program like the FAP can provide a higher level of therapeutic intensity and proximity to professional support, which is needed to help families feel more contained and supported in challenging the AN to a more extreme, but necessary, level than would otherwise be possible in typical outpatient administration of FBT.

Whilst this study has provided the first empirical explorations of the FAP that can highlight components of treatment important to a family admission for AN, it nonetheless possesses limitations that have implications for the utility of these findings. First, the sample size was small, particularly for the IPA. Whilst this size has been deemed empirically sufficient in previous IPA studies [27], a larger number of participants could have enabled more depth and breadth to the range of thematic categories identified. Second, all the authors had practise in FBT. Whilst the knowledge-base generated by this clinical experience likely helped to enable a deeper understanding of the treatment components and experiences that participants described in interviews, it also had the potential to influence the way in which data was analysed and interpreted. Precautions were adopted to try to accommodate for this limitation, including adopting a philosophical stance of inquiry that acknowledged the position and influences of the researchers as an active part of the empirical process [22, 24]; however, there nonetheless remained the likelihood that biases existed in the way that meaning was made from the analytical process. Notwithstanding these issues, the current study nonetheless offers important insights into the nature of the FAP, and can thus inform how future quantitative research regarding family-based hospital admission programs could be carried out to more directly investigate specific mechanisms of therapeutic change. In doing so, the avenues for treatment for individuals and families unlikely to respond successfully to FBT can continue to grow and be enhanced.

## Abbreviations

AN: Anorexia Nervosa
CHW: Children’s Hospital at Westmead
FAP: Family Admissions Program
FBT: Family Based Treatment
IPA: Interpretative Phenomenological Analysis

## References

[1] Lock J (2015). An update on evidence-based psychosocial treatments for eating disorders in children and adolescents. J Clin Child Adolesc Psychol.

[2] Lock J, Le Grange D. Treatment manual for anorexia nervosa: a family-based approach. 2nd ed. New York, NY: Guilford Press; 2012.

[3] Madden S, Miskovic-Wheatley J, Wallis A, Kohn M, Lock J, Le Grange D, Touyz SA (2015). Randomized controlled trial of in-patient treatment for anorexia nervosa in medically unstable adolescents. Psychol Med.

[4] Watson HJ, Bulik CM (2013). Update on the treatment of anorexia nervosa: review of clinical trials, practice guidelines and emerging interventions. Psychol Med.

[5] Le Grange D, Eisler I (2009). Family interventions in adolescent anorexia nervosa. Child Adolesc Psychiatr Clin N Am.

[6] Keel PK, Haedt A (2008). Evidence-based psychosocial treatments for eating problems and eating disorders. Journal of Clinical Child and Adolescent Psychology.

[7] Bulik CM, Berkman ND, Brownley KA, Sedway JA (2007). Lohr. Anorexia nervosa treatment: a systematic review of randomised control trials. Int J Eat Disord.

[8] Eisler I, Simic M, Russell GFM, Dare CA (2007). Randomised controlled treatment trial of two forms of family therapy in adolescent anorexia nervosa: a five-year follow-up. J Child Psychol Psychiatry.

[9] Le Grange D, Eisler I, Dare C, Russell GFM (1992). Evaluation of family treatments in adolescent anorexia nervosa: a pilot study. Int J Eat Disord.

[10] Szmukler G, Eisler I, Russell GFM, Dare C (1985). Anorexia nervosa, parental ‘expressed emotion’ and dropping out of treatment. Br J Psychiatry.

[11] Eisler I, Dare C, Hodes M, Russell GFM, Dodge E, Le Grange D (2000). Family therapy for anorexia nervosa: the results of a controlled comparison of two family interventions. J Child Psychol Psychiatry.

[12] Lock J, Agras S, Bryson S, Kraemer HA (2005). Comparison of short and long term family therapy for adolescent anorexia nervosa. Journal of American academic child and. Adolesc Psychiatry.

[13] Lock J, Couturier J, Bryson S, Agras S (2006). Predictors of drop-out and remission in family therapy for adolescent anorexia nervosa in a randomised control trial. Int J Eat Disord.

[14] Jewell T, Blessitt E, Stewart C, Simic M, Eisler I. Family therapy for child and adolescent eating disorders: a critical review. Fam Process. 2016;55:1–18. 10.1111/famp.12242 27543373

[15] Girz L, Robinson AL, Foroughe M, Jasper K, Boachie A (2013). Adapting family-based therapy to a day hospital programme for adolescents with eating disorders: preliminary outcomes and trajectories of change. J Fam Ther.

[16] Hoste R (2015). Incorporating family-based therapy principles into a partial hospitalization programme for adolescents with anorexia nervosa: challenges and considerations. J Fam Ther.

[17] Wallis A, Alford C, Hanson A, Titterton J, Madden S, Kohn M (2013). Innovations in Maudsley family-based treatment for anorexia nervosa at the Children's Hospital at Westmead: a family admission programme. J Fam Ther.

[18] Lock J, Le Grange D, Agras WS, Fitzpatrick KK, Jo B, Accurso E, Stainer M (2015). Can adaptive treatment improve outcomes in family-based therapy for adolescents with anorexia nervosa? Feasibility and treatment effects of a multi-site treatment study. Behav Res Ther.

[19] Ridgeway P (2001). ReStorying psychiatric disability. Learning from first person recovery narratives. Psychiatric Rehabilitation Journal.

[20] Denzin NK (1978). Sociological methods.

[21] Bekhet AK, Zauszniewski JA (2012). Methodological triangulation: an approach to understanding data. Nurse Researcher.

[22] Clandinin DJ, Connelly FM (2000). Narrative inquiry: experience and story in qualitative research.

[23] Pietkiewicz I, Smith JAA (2014). Practical guide to using interpretative phenomenological analysis in qualitative research psychology. Psychological Journal.

[24] Gergen M, Gergen K (2006). Narratives in action. Narrat Inq.

[25] Polkinghorne D (1995). Narrative configuration in qualitative analysis. Qualitative Studies in Education.

[26] Kirkpatrick H, Byrne CA (2009). Narrative inquiry: moving on from homelessness for individuals with a major mental illness. J Psychiatr Ment Health Nurs.

[27] Smith JA, Osborn M, Smith J (2008). Interpretative phenomenological analysis. Qualitative psychology: a practical guide to research methods.

[28] Ellison R, Rhodes P, Madden S, Miskovic J, Wallis A, Baillie A, Touyz S (2012). Do the components of manualized family-based treatment for anorexia nervosa predict weight gain?. Int J Eat Disord.

[29] Dare C, Eisler I, Colahan M, Crowther C, Senior R, Asen E (1995). The listening heart and the chi square: clinical and empirical perceptions in the family therapy of anorexia nervosa. J Fam Ther.

[30] Whitney J, Eisler I (2005). Theoretical and empirical models around caring for someone with an eating disorder: the reorganisation of family life and inter-personal maintenance factors. J Ment Health.

[31] White M, Epston D (1990). Narrative means to therapeutic ends.

[32] Gedo PM, Cohler BJ (1992). Session frequency, regressive intensity, and the psychoanalytic process. Psychoanal Psychol.

[33] Freedman N, Hoffenber JD, Vorus N, Frosch A (1999). The effectiveness of psychoanalytic psychotherapy: the role of treatment duration, frequency of sessions, and the therapeutic relationship. Journal of the American Psychonalytic Association.

[34] Rhodes P, Baillie A, Brown J, Madden S (2005). Parental efficacy in the family-based treatment of anorexia: preliminary development of the parents versus anorexia scale (PVA). Eur Eat Disord Rev.

[35] Heatherington L, Friedlander ML, Diamond GM, Escudero V, Pinsof WM (2015). 25 years of systemic therapies research: progress and promise. Psychother Res.

[36] Sly R, Morgan JF, Mountford VA, Lacey JH (2013). Predicting premature termination of hospitalised treatment for anorexia nervosa: the roles of therapeutic alliance, motivation and behaviour change. Eating Behaviours.

[37] Forsberg S, LoTempio E, Bryson S, Fitzpatrick KK, Le Grange D, Lock J (2013). Int J Eat Disord.

[38] Pereira T, Lock J, Oggins J (2006). Role of therapeutic alliance in family therapy for adolescent anorexia nervosa. Int J Eat Disord.

[39] Le Grange D, Lock J, Agras WS, Moye A, Bryson SW, Jo B (2012). Moderators and mediators of remission in family-based treatment and adolescent focused therapy for anorexia nervosa. Behav Res Ther.

